# Ghrelin, Amylin, Gastric Inhibitory Peptide and Cognition in Middle-Aged HIV-Infected and Uninfected Women: The Women’s Interagency HIV Study

**DOI:** 10.4172/2155-9562.1000413

**Published:** 2017-02-08

**Authors:** Samy I McFarlane, Michelle M Mielke, Anthony Uglialoro, Sheila M Keating, Susan Holman, Howard Minkoff, Howard A Crystal, Deborah R Gustafson

**Affiliations:** 1 Department of Medicine, Division of Endocrinology, State University of New York - Downstate Medical Center, Brooklyn, NY, USA; 2 Department of Health Sciences Research, Division of Epidemiology, and Department of Neurology Mayo Clinic, Rochester, MN, USA; 3 Empire Clinical Research Program (ECRIP) fellow, Department of Neurology, State University of New York - Downstate Medical Center, Brooklyn, NY, USA; 4 Blood Systems Research Institute, San Francisco, CA, USA; 5 Department of Medicine/STAR Program, State University of New York - Downstate Medical Center, Brooklyn, NY, USA; 6 Maimonides Hospital, Brooklyn, NY, USA; 7 Department of Obstetrics and Gynecology, State University of New York - Downstate Medical Center, Brooklyn, NY, USA; 8 Department of Neurology, State University of New York - Downstate Medical Center, Brooklyn, NY, USA; 9 Neuropsychiatric Epidemiology Unit, University of Gothenburg, Gothenburg, Sweden

**Keywords:** Cognition, Ghrelin, Amylin, Gastric Inhibitory Peptide (GIP), HIV, Women, Overweight, Obesity, Middle-aged

## Abstract

**Objective:**

To explore the gut-brain axis by examining gut hormone levels and cognitive test scores in women with (HIV+) and without (HIV−) HIV infection.

**Design/methods:**

Participants included 356 women (248 HIV+, 108 at risk HIV−) in the Brooklyn Women’s Interagency HIV Study (WIHS) with measured levels of ghrelin, amylin and gastric inhibitory peptide (GIP), also known as glucose-dependent insulinotropic polypeptide. Cross-sectional analyses using linear regression models estimated the relationship between gut hormones and Trails A, Trails B, Stroop interference time, Stroop word recall, Stroop color naming and reading, and Symbol Digit Modalities Test (SDMT) with consideration for age, HIV infection status, Wide Range Achievement Test score (WRAT), CD4 count, insulin resistance, drug use, and race/ethnicity.

**Results:**

Among women at mid-life with chronic (at least 10 years) HIV infection or among those at risk, ghrelin, amylin and GIP were differentially related to cognitive test performance by cognitive domain. Better performance on cognitive tests was generally associated with higher ghrelin, amylin and GIP levels. However, the strength of association varied, as did significance level by HIV status.

**Conclusion:**

Previous analyses in WIHS participants have suggested that higher BMI, waist, and WHR are associated with better cognitive function among women at mid-life with HIV infection. This study indicates that higher gut hormone levels are also associated with better cognition. Gut hormones may provide additional mechanistic insights regarding the association between obesity and Type 2 diabetes and cognition in middle-aged HIV+ and at risk HIV− women. In addition, measuring these hormones longitudinally would add to the understanding of mechanisms of actions of these hormones and their use as potential clinical tools for early identification and intervention on cognitive decline in this vulnerable population.

## Introduction

Survival with Human Immunodeficiency Virus (HIV) infection has been extended because of antiretroviral therapies (ART). Thus, HIV infection is becoming a chronic infection of aging, along with other aging-related conditions, such as Type 2 diabetes (T2D), cardiovascular diseases, and cognitive impairments. In many countries, the advent of ART has been accompanied by an increase in body mass index (BMI) that mirrors trends in the general population. Thus individuals with HIV infection are becoming more overweight and obese, which increases their risk for developing the aforementioned leading causes of disability and death in the United States and around the world, particularly of interest here, cognitive impairments [1–3].

The gut brain axis has been extensively implicated in human health. Hormones secreted by the gut have been shown to interact with the brain and regulate feeding behavior and energy balance [4]. Food intake behavior and energy homeostasis are strongly regulated by a complex system of humoral factors and neural structures constituting the gut-brain axis. To date, the only known peripherally produced and centrally acting peptide that stimulates food intake (orexigenic) is ghrelin, which is mainly synthesized in the stomach.

Ghrelin, discovered by Japanese researchers in 1999, is an orexigenic polypeptide (i.e., it stimulates the appetite and increases dietary intake), composed of 28 amino acids, and is secreted mainly by the P/DI cells lining the stomach fundus and, to a lesser degree, by various organs: intestine, pancreas, kidney, hypothalamus, and pituitary gland [5,6]. Ghrelin secretion increases under fasting conditions and falls after food intake. After gastrectomy, the concentration of ghrelin in plasma falls approximately 75%, which shows the importance of its secretion by the stomach [7,8].

Ghrelin may interact with leptin, which induces satiety at high levels, to regulate energy balance. Ghrelin is an example of the neurochemical overlap between reward and energy balance regulation systems, and the reward systems have been implicated in addictive behaviors such as compulsive overeating and drug dependence [9,10]. Ghrelin also appears to activate the cholinergic-dopaminergic reward link, which is associated with reward and motivated behavior, such as food searching. In the brain, ghrelin is produced by the arcuate nucleus and stimulates secretion of HGH from the anterior pituitary [7]. Ghrelin also acts on the level of the hypothalamus by stimulating biosynthesis and secretion of neuropeptide Y and Agouti-Related Protein [5,11]. Polymorphisms in the pro-ghrelin gene have been associated with obesity [10] and methamphetamine dependence, while polymorphisms in the ghrelin receptor gene have been associated with bulimia [12] and obesity [7,10,12,13].

Amylin, a 37 amino acid peptide, is co-secreted with insulin by pancreatic beta cells, either in response to glucose or sulfonylureas stimulation [14]. This is in contrast to ghrelin secretion, described above. Amylin, which is deficient in those with T2D, inhibits glucagon secretion, delays gastric emptying, and promotes satiety, thereby decreasing postprandial blood glucose [15–17]. Plasma amylin is positively associated with cognitive function [18–20]. Amylin treatment improves memory in AD mouse models [19]. However, amylin’s activities in cognition are impaired in the presence of T2D [20].

Gastric Inhibitory Peptide (GIP), also referred to as Glucose-dependent insulinotropic peptide, is an incretin, an intestinal peptide secreted by the gut in response to dietary intake of glucose, lipids and carbohydrates [21]. Incretins have been suggested to be a key link to obesity and physiological responses to today’s obesigenic environments. In fact, analogues of GIP have been developed to treat T2D. Some of these analogues cross the blood-brain barrier, are neuroprotective, activate the brain’s neuronal stem cells, and improve cognition. Receptors for GIP are expressed in neurons, and GIP is expressed and released as a transmitter by neurons [22]. GIP analogues such as DAla(2)GIP, enhance synaptic plasticity in the brain and reverse the beta amyloid induced impairment of synaptic plasticity in Alzheimer mouse models [23].

Today, according to PubMed, there are no published reports on the association between GIP and cognition or dementia in adults infected or not with HIV. Studies in mice are equivocal [24,25], however studies of GIP receptor knockout mice show impaired cognition [26–28]. In contrast, activation of GIP agonists has been shown to improve cognition in mouse models [29].

In the present study, we investigated whether levels of three gut hormones are differentially associated with cognitive performance in middle-aged women who are HIV+ vs HIV− and participate in the Women’s Interagency HIV Study (WIHS) [21,22]. Previous analyses in this same group of women have shown that higher levels of BMI and lower levels of blood leptin are associated with better cognitive function [30,31].

## Materials and Methods

The WIHS is an ongoing prospective study of HIV infection in women [32]. WIHS began in 1994 and enrolled 3766 women across six sites in San Francisco, Los Angeles, Chicago, Washington, DC, Brooklyn and the Bronx (New York). Across all sites, WIHS initially recruited 2054 HIV infected (HIV+) and 569 at-risk HIV uninfected (HIV−) women in 1994–1995 and an additional 737 HIV-infected and 406-HIV uninfected women in 2001–2002. The Brooklyn WIHS site has participated since the WIHS’ inception. Among the Brooklyn participants 356 (247 HIV+, 107 HIV−) had available cognitive tests and gut hormone measures. Written informed consent was provided by all Brooklyn WIHS participants via a human subject’s protocol that was approved by the SUNY Downstate Medical Center Institutional Review Board (protocol # 266921).

### Demographic measures

All demographic measures were self-reported. Race [32,33] was self-reported as white, Hispanic, African-American (AA), or ‘other’ (e.g., Native American/Alaskan, Asian/Pacific Islander) for all participants. Participants are also asked to report the current socioeconomic status, educational levels attained, smoking status, and use of marijuana, ‘crack’, cocaine, and heroin.

### Clinical measures

Anthropometric measures were conducted according to the NHANES III protocol, wearing light clothing, and included body weight (pounds), body height (inches), waist and hip circumferences (cm), and BMI (kg/m^2^) [23]. Body weight was recorded to the nearest 1.0 pound, and body height was measured to the nearest 1.0 inch. After conversion of body weight and height to metric units, BMI was calculated as kilograms per meter squared (kg/m^2^). Categories of BMI included ≥ 25 kg/m^2^ for overweight and obesity and ≥ 30 kg/m^2^ for obesity [34]. Waist and hip circumferences were measured to the nearest 0.5 cm. WHR was calculated as the ratio of waist to hip circumference. Central obesity was defined as WHR >0.80 or WC ≥ 88 cm [35].

Eight hour fasted blood samples were collected and total cholesterol levels were determined as previously described [26]. Systolic (SBP) and diastolic blood pressures (DBP) were recorded using a standardized protocol [36]. Hypertension was defined as either average measured SBP>140 mm Hg or DBP >90 mm Hg, self-reported hypertension, or use of antihypertensive medications. Previous myocardial infarction (MI) and T2D were self-reported [32,33].

### Biomarker analysis

Plasma samples, standards and controls were tested in duplicate using an active ghrelin ELISA, and active amylin and gastric inhibitory polypeptide (GIP) were measured by Luminex multiplex assay (Millipore, Billerica, MA). For active ghrelin, samples were tested undiluted and plates were prepared according to protocol and quantified using a 6-point standard curve ranging from 172 to 5500 ng/mL. Plates were read using a Molecular Devices Plate reader and Softmax Pro data analysis software. A 5-PL curve fit was used. Data analysis was performed using Softmax Pro 5.0. For amylin and GIP, samples, standards and controls were tested in duplicate and the assay was prepared according to protocol using a 7 point standard curve. Plates were read using the Bioplex 200 with Bioplex Manager (BioRad, Hercules, CA).

### HIV-related variables

Methods for determining HIV status, AIDS diagnosis, CD4 count, viral load, and duration of ART use were described previously [32,33].

### Cognitive tests

Cognitive tests (Trails A, Trails B, Stroop interference time, Stroop word recall, Stroop color naming and reading, Symbol Digit Modalities Test (SDMT) and the Wide Range Achievement Test (WRAT) were administered to all English-speaking WIHS participants during visits 21 to 24 (October 2004 to September 2006) as part of the WIHS core assessment; the Comalli-Kaplan Stroop was administered to a subgroup during visits 25–28, October 2006 to September 2008 (Table 1). These tests have been previously described [41]. Among participants who completed testing on multiple visits, and therefore have more than one score, only the first score was used. Times greater than 240 s were coded as 240 s, errors were recorded, but were not used to adjust interference times. For all cognitive tests, we used raw scores rather than normalized data.

### Inclusion criteria

We include all data collected by visit 28 among all Brooklyn WIHS participants, concluding in September 2008 for 356 participants (247 HIV+, 107 HIV−) with data available on both anthropometric and cognitive measures.

### Statistical analysis

Gut hormones were considered as continuous variables, except for ghrelin. Ghrelin was considered in quintiles due to measurement limits of the assay and one-fifth of samples in the lowest quintile having the same value. Linear regression analyses were used to examine associations between continuous or categorical gut hormones and cognitive test scores (time to completion) of Trails A, Trails B, SDMT score, Stroop interference, Stroop Color Naming, and Stroop Word Recall. Regression models were run separately for infected and uninfected women.

Several covariates were considered including: age, race, highest educational level attained, Wide Range Achievement Test (WRAT) score, HIV status, ART, CD4 count, CD4 nadir, prevalent DM, SBP, DBP, use of anti-hypertensive medications, use of exogenous insulin, blood cholesterol level, current smoking status, and use of marijuana, ‘crack’, cocaine, and/or heroin. Potential covariates were included if significant in age-adjusted models at a level of *p*<0.05. Given this significance level, final models included the following covariates: age, WRAT, race, exogenous insulin, use of antiretroviral therapy (ART) and any recreational drug use. Other HIV-related covariates evaluated, such as CD4 count or CD4 nadir, were not included because they were not associated with cognition or the gut hormones. In analyses of women who were HIV+, we also adjusted for HIV viral load. In sensitivity analyses of both HIV+ and HIV− women, we excluded drug users. STATA 12 was used for all statistical analyses. Results were considered statistically significant at *p*<0.05.

## Results

Both gut hormone and cognitive measures were available for 356 Brooklyn WIHS participants (213 HIV+ and 97 HIV− women). Demographic, anthropometric, and health characteristics are presented in Table 2. Based on average age (mean age 38.9 years), these women were not at risk for late-onset, aging-related cognitive impairments or dementias. HIV+ women were approximately 4 years older than HIV− women, however educational attainment, a key influencer of cognitive performance, did not differ between HIV+ and HIV− women. As previously reported, most participants were overweight or obese (≥ 25.0 kg/m^2^) and the frequency of central obesity was high.

**Figure 1:**
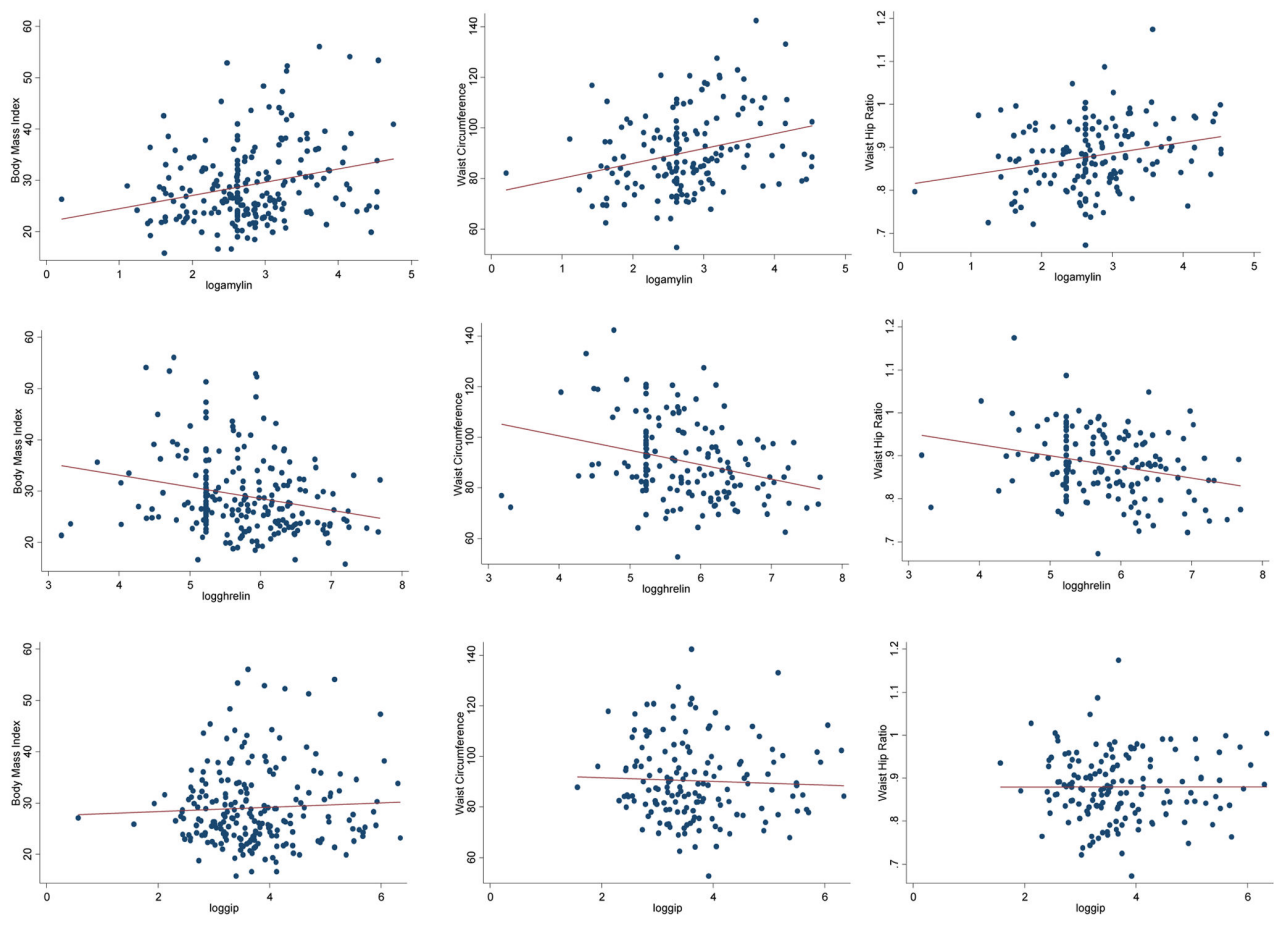
Correlation plots illustrating the association of anthropometric measures and gut hormones: The Women’s Interagency HIV Study (WIHS).

Correlations between anthropometric measures and gut hormones indicated modest associations in the directions expected for ghrelin and amylin (Table 3 and Figure 1). Notably the correlation coefficients were positive for anthropometric measures and amylin, and negative for ghrelin. No correlation was observed for GIP and anthropometric measures. In addition, there were lower average levels of amylin, WHR and BMI (*p*<0.10) among HIV+ women compared to uninfected women WHR was higher among HIV+ women. Neither ghrelin nor GIP was associated with HIV infection status.

Associations between gut hormones and cognitive test scores in linear regression models revealed an inverse association between amylin and Trails A in HIV+ and HIV− participants after multivariate adjustment (Model 2) (Table 4). An inverse association was also observed for GIP among HIV+ women. Ghrelin was inversely associated with Trails A and Stroop Color Naming time among HIV− women. Ghrelin was also associated with Stroop Word Reading time in HIV+ women, as was GIP. In summary, higher gut hormone levels were associated with better cognition.

## Discussion

Among women at mid-life with HIV infection for at least 10 years, or among those at risk, better performance on cognitive tests was generally associated with higher gut hormone levels. However, the strength of association varied, as did significance level by HIV status. To our knowledge, there are no published reports on these gut hormones in association with cognition in HIV-infection.

The importance of understanding the association of gut hormones with cognition, originates from published associations between high BMI and obesity or metabolic syndromes with cognition and dementia among HIV uninfected population samples [1,43–45]. High mid-life BMI [2,43,44] central obesity (measured as waist circumference or WHR), [3,45] and T2D [46] have been shown to increase risk for Alzheimer’s Disease (AD) in uninfected populations. Simple anthropometric measures reflect different aspects of body composition, and, at best, are crude indicators of the body’s metabolism in response to energy intake. A high BMI is reasonably correlated with whole body amount of adipose tissue and higher energy intake in healthy HIV-negative adults [47]. Going further to assess gut hormone associations among those with or at risk for HIV infection, may enhance our understanding of mechanisms underlying this association. High BMI and WC during mid-life are also related to other vascular risk factors, such as T2D [48], hypertension, and hyperlipidemia [49], which increase risk for cognitive impairments and dementia in non-HIV populations.

Amylin is interesting for cognitive brain health for several reasons. Potential key roles of amylin in cognition and AD are related to: 1) its co-secretion with insulin from pancreatic beta-cells to regulate postprandial glycemia; 2) its role in the development of T2D (amylin is deficient in T2D); 3) T2D being a risk factor for AD and vascular forms of dementia; and 4) amylin being pancreatic islet amyloid polypeptide (PIAPP), in comparison and contrast to the amyloid precursor protein (APP) that is differentially spliced to form the fragments that form amyloid-beta (Ab) that deposits in the aging brain and has been touted as the underlying neuropathologic molecule responsible for Alzheimer’s Disease [50,51]. Some data suggest that amylin may play a vital role at the interface between peripheral and neurodegenerative disorders, and that amylin and Aβ interact in the brain [51]. Evidence to support the latter includes PIAPP being present in human cerebrospinal fluid (CSF), synthetic PIAPP promoting Aβ oligomerization *in vitro* and endogenous IAPP localizing to Aβ oligomers and plaques [52]. While higher levels of blood amylin are associated with higher BMI, lower blood levels of amylin have been observed in adults with more severe forms of cognitive impairment, such as dementia [18]. This is congruent with the low BMI and low leptin levels observed cross-sectionally in those with dementia, and that cognition is enhanced with the amylin analog, pramlintide [19]. Similar to other reports in HIV− samples that relate anthropometric measures to gut hormone levels, our findings demonstrate that higher amylin levels were associated with higher levels of overweight and obesity [53].

Ghrelin may also improve cognition [54]. Executive function and speed of information processing are the cognitive domains most commonly associated with these hormonal measures. Ghrelin plays a role in multiple physiological processes including appetite regulation, metabolism and, more recently, dendritic spine architecture, long-term potentiation and cognition [55]. Small-molecule ghrelin receptor agonists readily cross the blood brain barrier and elicit pro-cognitive effects in recognition and spatial learning and memory tests [55]. Due to its ubiquitous and diverse character, ghrelin is also of interest as a Zeitgeber referring to environmental cues that resets the body’s circadian rhythm [56]. Meal times and the hormones associated with them are a subset of these internal cues and may help to synchronize circadian rhythms. Circadian rhythms are disrupted in AD [56]. In addition, declining circulating ghrelin has shown association with reduced appetite, reduced hippocampal neurogenesis and synaptic plasticity, weaker feeding-related zeitgeber, memory impairments, weight loss, and as aforementioned, disrupted circadian rhythms. All of these symptoms are associated with cognitive impairments and AD [56].

As shown in HIV− samples, ghrelin is inversely associated with BMI. However, in contrast with one published report on differences in several metabolic hormones and higher ghrelin levels in HIV+ versus HIV− controls, we observed no difference in mean ghrelin levels by HIV status [57].

GIP was not associated with cognitive test scores or HIV status, nor was it correlated with anthropometric measures. This may indicate agreement with the observed performance of GIP analogs versus native GIP in animal models in relation to cognition [27]. There is one published study on GIP in HIV infection, comparing those with *versus* without glucose intolerance [58]. GIP was associated with insulin secretion rates irrespective of glucose intolerance status. GIP has been associated with some cognitive outcome measures, primarily in animal models [24,26,27,29]. Results of studies on GIP or other incretins and their analogs in cognition and AD have not been reported.

The influence of ART on cognition and overall health in HIV is inconsistent depending on the severity of cognitive outcome being assessed and age of the infected. Data suggest no difference in the proportion of individuals with HIV-associated cognitive disorder (HAND) in the pre- versus post-ART eras [59]; however, the prevalence of AIDS dementias, the most severe form of impairment, has fallen precipitously concurrent with optimization of medication regimens and better care overall [60]. As HIV-infected populations survive to older ages, they may be at risk for more severe cognitive impairments and age-related dementias, such as sporadic AD. This would be a new phenomenon, but is speculative at this time. ART may also have cardiovascular side effects, such as atherosclerosis [61,62], DM [63] and hypertension [64], even in HIV-infected children [65]. These cardiovascular factors are related to risk for AD in populations without HIV infection; and cardiovascular risk factors are associated with worse cognition in persons with HIV [66,67]. Thus, this begs the question of whether we are setting the stage for a form of iatrogenic AD in HIV-infected populations. While the positive influence of ART on cognition is clear [68]; there are several controversial studies providing data that suggest a negative influence [69]. Some studies show that discontinuing ART is associated with improved performance on cognitive tests [70]; and it has been speculated that certain ART regimens are deleterious for cognition [71]. Even so, alterations in gut tissue hormones, adipose tissue, adipose tissue distribution, adipose tissue hormones and/or lipid metabolism observed in HIV infection [31,72], may also create an altered vascular, metabolic and/or hormonal milieu that is undesirable for the brain [73].

This is a large study of gut hormones and cognition in women with and without HIV infection. Strengths include the large multi-ethnic participant sample, and three gut hormone measures that have been evaluated to a limited extent in uninfected elderly samples in association with cognition. The primary limitations include a relatively few battery of cognitive tests, the existence of other unmeasured gut hormones of potential interest (e.g. GLP-1^55^), a cross-sectional study design and analysis, and the average age of participants being 39 years, making it perhaps difficult to detect influencers of cognitive function. In addition, due to multiple comparisons, and relatively high p-values, one must also consider risk for false discoveries. Our analyses were not adjusted for multiple comparisons. However, since this is one of the first reports of common gut hormones in relation to cognition in HIV, we chose an empirical data analysis approach. Not adjusting for multiple comparisons is preferable because it leads to fewer errors of interpretation and follows more closely, untainted natural observations of association [74]. Of note, this investigation of gut hormones and cognition represents a site-specific (Brooklyn only) sub-study of middle-aged women within the greater WIHS multi-center network. While WIHS has collected much information on a variety of factors and biomarkers for over 20 years as a result of its interdisciplinary nature, due to the exclusive availability of gut hormone measures at one point in time at one site, our analysis capacity to integrate all additional WIHS data is limited.

In summary, these data suggest the need for continued follow up of these women to determine mid-life and late-life effects of gut hormones, dietary factors and overweight and obesity on cognition and dementia in HIV.

## Figures and Tables

**Table 1:** Cognitive tests administered in the WIHS and corresponding cognitive domains measured [41,42].

Cognitive Domain	Test
**Executive Function**	Trails A, Trails B
	Stroop Interference [37,38]
**Speed of Information Processing**	Symbol Digit Modalities Test (SDMT)* [39,40]
	Stroop Color Naming and Reading
**Learning and Memory**	Stroop Word Recall

* The SDMT score is the number of correct items in 90 s, all other test scores are times with lesser time indicating better performance

**Table 2:** Characteristics of WIHS participants with gut hormone measures.

	ALL (n=356)		HIV+ (n=248)		HIV− (n=108)		
Characteristic	N	Mean (SD)/n (%)	N	Mean (SD)/n (%)	N	Mean (SD)/n (%)	*p*-value
Age	356	38.9 (9.1)	248	40.0 (8.5)	108	36.5 (9.9)	<0.001
Race	356		248		108		0.46
White		31 (8.7%)		24 (9.7%)		7 (6.5%)	
African American (AA)		282 (79.1%)		194 (78.2%)		88 (81.5%)	
Non-white, non-AA Hispanic		34 (9.6%)		23 (9.3%)		11 (10.2%)	
Other		9 (2.6%)		7 (2.8%)		2 (1.8%)	
Highest education	355		1194		491		0.571
Grades 7–11		126 (35.5%)		428 (35.9%)		158 (32.2%)	
Completed HS		127 (35.8%)		377 (31.6%)		162 (33.0%)	
Some college		86 (24.2%)		305 (25.5%)		131 (26.7%)	
4 years degree		14 (3.9%)		62 (5.2%)		32 (6.5%)	
attend/complete grad school		2 (0.6%)		22 (1.8%)		8 (1.6%)	
CD4 count			245	27,537 (140,597)			
Viral load			246	518.5 (322.7)			
GIP (pg/ml)	355	69.1 (87.4)	248	69.4 (82.5)	107	68.4 (98.3)	0.919
Ghrelin (pg/ml)	356	365.5 (223.6)	248	359.7 (316.6)	108	379.0 (367.9)	0.616
Amylin (pg/ml)	347	24.4 (51.4)	242	20.5 (20.2)	105	33.4 (87.9)	0.031
Diabetes mellitus	313	15 (4.8%)	218	9 (4.1%)	95	6 (6.3%)	0.4
BMI	351	29.2 (7.9)	246	28.8 (7.3)	105	30.3 (9.1)	0.089
WHR	266	0.88 (0.08)	181	0.89 (0.08)	85	0.85 (0.07)	0.0001
Marijuana use since last visit	356	64 (18.0%)	248	41 (16.5%)	108	23 (21.3%)	0.295
Any indicator of hypertension*	356	129 (36.2%)	248	95 (38.3%)	108	34 (31.5%)	0.232
Total Cholesterol	356	176.7 (26.39)	248	175.4 (37.3)	108	179.8 (34.1)	0.296

* Either SBP>=140, DBP>=90, self-reported hypertension, or taking anti-hypertensive medication

**Table 3:** Pearson correlations between anthropometric measures and gut hormones. The Women’s Interagency HIV Study (WIHS).

	BMI					Waist					WHR				
		Crude		Age-adjusted					Age-adjusted			crude		age-adjusted	
	n	r	*p*-value	r	*p*-value	n	r	*p*-value	r	*p*-value	n	r	*p*-value	r	*p*-value
Log amylin	342	0.230	<0.0001	0.229	<0.0001	260	0.297	<0.0001	0.276	<0.0001	258	0.276	<0.0001	0.276	<0.0001
Log ghrelin	351	−0.182	<0.001	−0.181	<0.001	268	−0.202	<0.001	−0.2	0.001	266	−0.119	0.053	−0.116	0.06
Log GIP	351	−0.028	0.602	−0.050	0.417	267	−0.046	0.455	−0.059	0.344	265	0.059	0.342	0.024	0.699

**Table 4:** Gut-brain hormones in association with cognitive test score, by HIV infection status: the Women’s Interagency HIV Study.

	All			HIV+			HIV−		
**Trails A**									
**Log Amylin - Continuous**									
Model 1	347	−2.85 (−4.90, −0.79)	0.007	242	−2.83 (−5.64, −0.04)	0.047	105	−2.03 (−4.83, 0.78)	0.156
Model 2	299	−2.44 (−4.74, −0.13)	0.038	211	−2.64 (−5.67, 0.40)	0.089	88	−1.78 (−5.10, 1.53)	0.287
**Log GIP - Continuous**									
Model 1	355	−1.28 (−2.98, 0.42)	0.141	248	−2.22 (−4.43, −0.02)	0.048	107	0.64 (−1.82, 3.10)	0.608
Model 2	307	−1.69 (−3.45, 0.06)	0.058	217	−2.41 (−4.67, −0.14)	0.037	90	0.24 (−2.42, 2.90)	0.859
**Log ghrelin – CATEGORICAL**									
Model 1									
Tertile 1 (low)	356	0 (ref)		248	0 (ref)		108	0 (ref)	
Tertile 2		0.38 (−3.59, 4.35)	0.851		3.42 (−1.64, 8.48)	0.184		−7.03 (−12.54, −1.51)	0.013
Tertile 3		−1.49 (−5.30, 2.33)	0.440		2.55 (−2.33, 7.43)	0.304		−10.7 (−15.94, −5.46)	<0.001
Model 2									
Tertile 1 (low)	308	0 (ref)		217	0 (ref)		91	0 (ref)	
Tertile 2		0.52 (−3.69, 4.73)	0.808		3.23 (−2.17, 8.63)	0.239		−6.94 (−12.88, −0.99)	0.023
Tertile 3		−1.49 (−5.49, 2.52)	0.465		2.49 (−2.65, 7.63)	0.340		−10.5 (−16.03, −4.96)	<0.001
**Trails B**									
**Log Amylin - Continuous**									
Model 1	346	−0.83 (−7.49, 5.84)	0.808	241	−3.11 (−12.41, 6.20)	0.511	105	3.03 (−5.40, 11.46)	0.478
Model 2	298	2.34 (−4.87, 9.55)	0.524	210	0.09 (−9.51, 9.69)	0.985	88	5.67 (−4.47, 15.82)	0.269
**Log GIP - Continuous**									
Model 1	354	−1.23 (−6.72, 4.26)	0.659	247	−4.05 (−11.33, 3.24)	0.275	107	4.14 (−3.25, 11.53)	0.269
Model 2	306	−2.32 (−7.81, 3.17)	0.406	216	−4.58 (−11.72, 2.57)	0.208	90	2.6 (−5.67, 10.87)	0.533
**Log ghrelin - Categorical**									
Model 1									
Tertile 1 (low)	355	0 (ref)		247	0 (ref)		108	0 (ref)	
Tertile 2		3.84 (−8.94, 16.63)	0.555		10.8 (−5.83, 27.43)	0.202		−11.91 (−29.69, 5.87)	0.187
Tertile 3		0.9 (−11.35, 13.14)	0.886		8.9 (−7.06, 24.87)	0.273		−17.23 (−34.11, −0.34)	0.046
Model 2									
Tertile 1 (low)	307	0 (ref)		216	0 (ref)		91	0 (ref)	
Tertile 2		4.82 (−8.31, 17.95)	0.471		10.05 (−6.84, 26.94)	0.242		−10.13 (−30.09, 9.82)	0.316
Tertile 3		−0.64 (−13.08, 11.80)	0.919		6.3 (−9.71, 22.31)	0.439		−16.78 (−35.34, 1.78)	0.076
**Digit Symbol**									
**Log Amylin - Continuous**									
Model 1	342	0.86 (−0.64, 2.36)	0.259	238	1.09 (−0.87, 3.06)	0.274	104	0.07 (−2.27, 2.40)	0.956
Model 2	295	−0.01 (−1.62, 1.61)	0.993	208	0.43 (−1.61, 2.47)	0.678	87	−0.85 (−3.57, 1.87)	0.536
**Log GIP - Continuous**									
Model 1	350	0.16 (−1.08, 1.40)	0.802	244	0.64 (−0.92, 2.19)	0.423	106	−0.76 (−2.78, 1.26)	0.460
Model 2	303	0.46 (−0.78, 1.69)	0.466	214	0.57 (−0.96, 2.11)	0.463	89	0.13 (−2.03, 2.29)	0.908
**Log ghrelin - Categorical**									
Model 1									
Tertile 1 (low)	351	0 (ref)		244	0 (ref)		107	0 (ref)	
Tertile 2		0.72 (−2.17, 3.61)	0.624		−1.18 (−4.76, 2.39)	0.515		5.46 (0.63, 10.29)	0.027
Tertile 3		1.3 (−1.49, 4.08)	0.360		0.1 (−3.37, 3.57)	0.954		3.98 (−0.56, 8.51)	0.085
Model 2									
Tertile 1 (low)	304	0 (ref)		214	0 (ref)		90	0 (ref)	
Tertile 2		1.03 (−1.92, 3.99)	0.492		−0.58 (−4.22, 3.06)	0.753		5.61 (0.44, 10.79)	0.034
Tertile 3		1.74 (−1.07, 4.55)	0.223		0.62 (−2.85, 4.10)	0.724		4.37 (−0.40, 9.13)	0.072
**Stroop - Color naming time**									
**Log Amylin - Continuous**									
Model 1	307	−0.09 (−2.68, 2.49)	0.944	212	0.07 (−3.36, 3.50)	0.968	95	0.18 (−3.65, 4.00)	0.926
Model 2	268	−0.17 (−3.01, 2.68)	0.908	186	0.33 (−3.26, 3.91)	0.858	82	−0.5 (−5.04, 4.03)	0.826
**Log GIP - Continuous**									
Model 1	314	−2.04 (−4.10, 0.01)	0.052	217	−2.88 (−5.50, −0.26)	0.031	97	−0.35 (−3.57, 2.86)	0.828
Model 2	275	−1.79 (−3.88, 0.30)	0.093	191	−2.27 (−4.92, 0.38)	0.093	84	−0.68 (−3.99, 2.64)	0.686
**Log ghrelin - Categorical**									
Model 1									
**Tertile 1 (Low)**	315	0 (ref)		217	0 (ref)		98	0 (ref)	
Tertile 2		0.11 (−4.68, 4.90)	0.964		2.05 (−4.10, 8.19)	0.512		−4.1 (−11.38, 3.19)	0.267
Tertile 3		−5.48 (−10.07, −0.89)	0.019		−3.78 (−9.64, 2.09)	0.205		−9.45 (−16.47, −2.44)	0.009
Model 2									
**Tertile 1 (Low)**	276	0 (ref)		191	0 (ref)		85	0 (ref)	
Tertile 2		−0.51 (−5.43, 4.42)	0.839		−0.66 (−7.00, 5.68)	0.838		−2.27 (−9.76, 5.22)	0.548
Tertile 3		−6.42 (−11.12, −1.72)	0.008		−5.8 (−11.79, 0.19)	0.057		−8.76 (−15.95, −1.57)	0.018
**Stroop - Word Read time**									
**Log Amylin - Continuous**									
Model 1	308	−0.38 (−2.53, 1.78)	0.731	213	−1.51 (−4.26, 1.24)	0.281	95	2.14 (−1.39, 5.67)	0.232
Model 2	269	−0.93 (−3.16, 1.31)	0.416	187	−1.88 (−4.58, 0.81)	0.169	82	2.32 (−1.74, 6.38)	0.259
**Log GIP - Continuous**									
Model 1	315	−0.41 (−2.13, 1.30)	0.636	218	−1.55 (−3.66, 0.55)	0.147	97	2.07 (−0.87, 5.01)	0.166
Model 2	276	−0.48 (−2.11, 1.16)	0.565	192	−1.29 (−3.28, 0.70)	0.203	84	1.37 (−1.56, 4.31)	0.355
**Log ghrelin - Categorical**									
Model 1									
Tertile 1 (low)	316	0 (ref)		218	0 (ref)		98	0 (ref)	
Tertile 2		0.82 (−3.13, 4.78)	0.683		1.56 (−3.33, 6.44)	0.53		−0.96 (−7.81, 5.88)	0.781
Tertile 3		−5.46 (−9.26, −1.66)	0.005		−5.14 (−9.82, −0.45)	0.032		−6.28 (−12.87, 0.31)	0.062
Model 2									
Tertile 1 (low)	277	0 (ref)		192	0 (ref)		85	0 (ref)	
Tertile 2		1.32 (−2.51, 5.14)	0.498		0.32 (−4.40, 5.03)	0.894		3.21 (−3.46, 9.89)	0.341
Tertile 3		−5.36 (−9.02, −1.70)	0.004		−5.79 (−10.27, −1.31)	0.012		−4.91 (−11.32, 1.50)	0.132
**Stroop - Interference time**									
**Log Amylin - Continuous**									
Model 1	302	−0.47 (−4.83, 3.89)	0.832	208	−1.19 (−6.79, 4.42)	0.677	94	−0.26 (−7.47, 6.96)	0.944
Model 2	265	−3.88 (−8.48, 0.73)	0.099	184	−2.93 (−8.50, 2.64)	0.301	81	−7.45 (−16.14, 1.22)	0.091
**Log gip - Continuous**									
Model 1	309	−2.59 (−6.04, 0.86)	0.140	213	−3.98 (−8.22, 0.27)	0.066	96	0.27 (−5.75, 6.29)	0.930
Model 2	272	−3.41 (−6.74, −0.07)	0.046	189	−4.07 (−8.11, −0.03)	0.049	83	−2.59 (−8.94, 3.76)	0.419
**Log ghrelin - Categorical**									
Model 1									
**Tertile 1 (Low)**	310	0 (ref)		213	0 (ref)		97	0 (ref)	
Tertile 2		1.91 (−6.23, 10.04)	0.645		5.83 (−4.21, 15.87)	0.254		−5.98 (−20.13, 8.16)	0.403
Tertile 3		−6.31 (−14.14, 1.52)	0.114		−4.55 (−14.20, 5.10)	0.353		−9.9 (−23.53, 3.73)	0.153
Model 2									
**Tertile 1 (Low)**	273	0 (ref)		189	0 (ref)		84	0 (ref)	
Tertile 2		4.83 (−3.20, 12.87)	0.237		7.17 (−2.63, 16.97)	0.151		−0.3 (−15.40, 14.80)	0.969
Tertile 3		−4.47 (−12.18, 3.25)	0.255		−4.09 (−13.42, 5.24)	0.388		−5.19 (−19.71, 9.32)	0.478

## Abbreviations

AIDS: Acquired Immunodeficiency Syndrome
AA: African American
AD: Alzheimer’s Disease
ART: Antiretroviral Therapies
BMI: Body Mass Index
cm: Centimeter
DBP: Diastolic Blood Pressure
ELISA: Enzyme-Linked Immunosorbent Assay
GIP: Gastric Inhibitory Peptide
GIP: Glucose-Dependent Insulinotropic Polypeptide
HIV: Human Immunodeficiency Virus
HAND: HIV-Associated Neurocognitive Disorder
HIV+: HIV Infected
kg: Kilograms
m: Meter
ml: Milliliter
HIV−: Not HIV Infected
pg: Picograms
SDMT: Symbol Digit Modalities Test
SBP: Systolic Blood Pressure
T2D: Type 2 Diabetes
WC: Waist Circumference
WHR: Waist-to-Hip Ratio
WRAT: Wide Range Achievement Test
WIHS: Women’s Interagency HIV Study
5-PL: 5-Parameter Logistic
MI: Myocardial Infarction

## References

[1] Whitmer RA, Gunderson EP, Quesenberry CP, Zhou J, Yaffe K (2007). Body mass index in midlife and risk of Alzheimer disease and vascular dementia. Curr Alzheimer Res.

[2] Fitzpatrick AL, Kuller LH, Lopez OL, Diehr P, O’Meara ES (2009). Midlife and late-life obesity and the risk of dementia: Cardiovascular health study. Arch Neurol.

[3] Gustafson DR, Bäckman K, Waern M, Ostling S, Guo X (2009). Adiposity indicators and dementia over 32 years in Sweden. Neurology.

[4] Wisser AS, Habbel P, Wiedenmann B, Klapp BF, Mönnikes H (2010). Interactions of gastrointestinal peptides: Ghrelin and its anorexigenic antagonists. Int J Pept.

[5] Kojima M, Hosoda H, Date Y, Nakazato M, Matsuo H (1999). Ghrelin is a growth-hormone-releasing acylated peptide from stomach. Nature.

[6] Kojima M, Kangawa K (2001). Ghrelin: a novel growth-hormone releasing peptide. Nihon Rinsho.

[7] Kroemer NB, Krebs L, Kobiella A, Grimm O, Pilhatsch M (2013). Fasting levels of ghrelin covary with the brain response to food pictures. Addict Biol.

[8] Perboni S, Inui A (2010). Appetite and gastrointestinal motility: Role of ghrelin-family peptides. Clin Nutr.

[9] Perello M, Scott MM, Sakata I, Lee CE, Chuang JC (2012). Functional implications of limited leptin receptor and ghrelin receptor co-expression in the brain. J Comp Neurol 2012.

[10] Schellekens H, Dinan TG, Cryan JF (2013). Ghrelin at the interface of obesity and reward. Vitam Horm.

[11] Di Vito L, Broglio F, Benso A, Gottero C, Prodam F (2002). The GH-releasing effect of ghrelin, a natural GH secretagogue, is only blunted by the infusion of exogenous somatostatin in humans. Clin Endocrinol (Oxf).

[12] Leggio L (2010). Role of the ghrelin system in alcoholism: Acting on the growth hormone secretagogue receptor to treat alcohol-related diseases. Drug News Perspect.

[13] Panagopoulos VN, Ralevski E (2014). The role of ghrelin in addiction: A review. Psychopharmacology (Berl).

[14] Moore CX, Cooper GJ (1991). Co-secretion of amylin and insulin from cultured islet beta-cells: modulation by nutrient secretagogues, islet hormones and hypoglycemic agents. Biochem Biophys Res Commun.

[15] Xue B, Zemel MB (2001). Agouti signaling protein stimulates islet amyloid polypeptide (amylin) secretion in pancreatic beta-cells. Exp Biol Med (Maywood).

[16] Yates SL, Burgess LH, Kocsis-Angle J, Antal JM, Dority MD (2000). Amyloid beta and amylin fibrils induce increases in proinflammatory cytokine and chemokine production by THP-1 cells and murine microglia. J Neurochem.

[17] Zhang S, Liu H, Chuang CL, Li X, Au M (2014). The pathogenic mechanism of diabetes varies with the degree of overexpression and oligomerization of human amylin in the pancreatic islet beta cells. FASEB J.

[18] Adler BL, Yarchoan M, Hwang HM, Louneva N, Blair JA (2014). Neuroprotective effects of the amylin analogue pramlintide on Alzheimer’s disease pathogenesis and cognition. Neurobiol Aging.

[19] Mietlicki-Baase EG (2016). Amylin-mediated control of glycemia, energy balance and cognition. Physiol Behav.

[20] Qiu WQ, Li H, Zhu H, Scott T, Mwamburi M (2014). Plasma amylin and cognition in diabetes in the absence and the presence of insulin treatment. J Diabetes Metab.

[21] Burcelin R (2005). The incretins: A link between nutrients and well-being. Br J Nutr.

[22] Holscher C (2010). Incretin analogues that have been developed to treat type 2 diabetes hold promise as a novel treatment strategy for Alzheimer’s disease. Recent Pat CNS Drug Discov.

[23] Faivre E, Hölscher C (2013). Neuroprotective effects of D-Ala(2)GIP on Alzheimer’s disease biomarkers in an APP/PS1 mouse model. Alzheimers Res Ther.

[24] Irwin N, Montgomery IA, Flatt PR (2012). Evaluation of the long-term effects of gastric inhibitory polypeptide-ovalbumin conjugates on insulin resistance, metabolic dysfunction, energy balance and cognition in high-fat-fed mice. Br J Nutr.

[25] Porter D, Faivre E, Flatt PR, Holscher C, Gault VA (2012). Actions of incretin metabolites on locomotor activity, cognitive function and *in vivo* hippocampal synaptic plasticity in high fat fed mice. Peptides.

[26] Faivre E, Gault VA, Thorens B, Holscher C (2011). Glucose-dependent insulinotropic polypeptide receptor knockout mice are impaired in learning, synaptic plasticity and neurogenesis. J Neurophysiol.

[27] Faivre E, Hamilton A, Hölscher C (2012). Effects of acute and chronic administration of GIP analogues on cognition, synaptic plasticity and neurogenesis in mice. Eur J Pharmacol.

[28] Lennox R, Moffett RC, Porter DW, Irwin N, Gault VA (2015). Effects of glucose-dependent insulinotropic polypeptide receptor knockout and a high-fat diet on cognitive function and hippocampal gene expression in mice. Mol Med Rep.

[29] Porter DW, Irwin N, Flatt PR, Holscher C, Gault VA (2011). Prolonged GIP receptor activation improves cognitive function, hippocampal synaptic plasticity and glucose homeostasis in high-fat fed mice. Eur J Pharmacol.

[30] Gustafson DR, Mielke MM, Keating SA, Holman S, Minkoff H (2015). Leptin, adiponectin and cognition in middle-aged HIV-infected and uninfected women. The Brooklyn Women’s Interagency HIV Study. J Gerontol Geriatric Res.

[31] Gustafson DR, Mielke MM, Tien PC, Valcour V, Cohen M (2013). Anthropometric measures and cognition in middle-aged HIV-infected and uninfected women. The Women’s Interagency HIV Study. J Neurovirol.

[32] Bacon MC, von Wyl V, Alden C, Sharp G, Robison E (2005). The women’s interagency HIV study: An observational cohort brings clinical sciences to the bench. Clin Diagn Lab Immunol.

[33] Barkan SE, Melnick SL, Preston-Martin S, Weber K, Kalish LA (1998). The women’s interagency HIV study. WIHS collaborative study group. Epidemiology.

[34] (1989). Diet and health: Implications for reducing chronic disease risk.

[35] Croft JB, Keenan NL, Sheridan DP, Wheeler FC, Speers MA (1995). Waist-to-hip ratio in a biracial population: Measurement, implications and cautions for using guidelines to define high risk for cardiovascular disease. J Am Diet Assoc.

[36] Mansoor A, Althoff K, Gange S, Anastos K, Dehovitz J (2009). Elevated NT-pro-BNP levels are associated with comorbidities among HIV-infected women. AIDS Res Hum Retroviruses.

[37] Stroop J (1935). Studies of interference in serial verbal reaction. J Exp Psychol.

[38] Comalli PE, Wapner S, Werner H (1962). Interference effects of stroop color-word test in childhood, adulthood and aging. J Genet Psychol.

[39] Smith A (1982). Symbol digit modalities test (SDMT). Manual (revised).

[40] Lezak M, Howieson DB, Loring DW (2004). Neuropsychological assessment.

[41] Crystal HA, Weedon J, Holman S, Manly J, Valcour V (2011). Associations of cardiovascular variables and HAART with cognition in middle-aged HIV-infected and uninfected women. J Neurovirol.

[42] Manly JJ, Smith C, Crystal HA, Richardson J, Golub ET (2011). Relationship of ethnicity, age, education and reading level to speed and executive function among HIV+ and HIV− women: The women’s interagency HIV study (WIHS) Neurocognitive sub study. J Clin Exp Neuropsychol.

[43] Kivipelto M, Ngandu T, Fratiglioni L, Viitanen M, Kåreholt I (2005). Obesity and vascular risk factors at midlife and the risk of dementia and Alzheimer disease. Arch Neurol.

[44] Whitmer RA, Gunderson EP, Barrett-Connor E, Quesenberry CP, Yaffe K (2005). Obesity in middle age and future risk of dementia: A 27 year longitudinal population based study. BMJ.

[45] Whitmer RA, Gustafson DR, Barrett-Connor E, Haan MN, Gunderson EP (2008). Central obesity and increased risk of dementia more than three decades later. Neurology.

[46] Xu W, Qiu C, Gatz M, Pedersen NL, Johansson B (2009). Mid- and late-life diabetes in relation to the risk of dementia: A population-based twin study. Diabetes.

[47] Shah NR, Braverman ER (2012). Measuring adiposity in patients: The utility of body mass index (BMI), percent body fat and leptin. PLoS One.

[48] Capeau J, Bouteloup V, Katlama C, Bastard JP, Guiyedi V (2012). Ten-year diabetes incidence in 1046 HIV-infected patients started on a combination antiretroviral treatment. AIDS.

[49] Breteler MM, Bots ML, Ott A, Hofman A (1998). Risk factors for vascular disease and dementia. Haemostasis.

[50] Jackson K, Barisone GA, Diaz E, Jin LW, DeCarli C (2013). Amylin deposition in the brain: A second amyloid in Alzheimer disease?. Ann Neurol.

[51] Lutz TA, Meyer U (2015). Amylin at the interface between metabolic and neurodegenerative disorders. Front Neurosci.

[52] Fawver JN, Ghiwot Y, Koola C, Carrera W, Rodriguez-Rivera J (2014). Islet amyloid polypeptide (IAPP): a second amyloid in Alzheimer’s disease. Curr Alzheimer Res.

[53] Lutz TA (2012). Control of energy homeostasis by amylin. Cell Mol Life Sci.

[54] Kunath N, van Groen T, Allison DB, Kumar A, Dozier-Sharpe M (2015). Ghrelin agonist does not foster insulin resistance but improves cognition in an Alzheimer’s disease mouse model. Sci Rep.

[55] Atcha Z, Chen WS, Ong AB, Wong FK, Neo A (2009). Cognitive enhancing effects of ghrelin receptor agonists. Psychopharmacology (Berl).

[56] Kent BA (2014). Synchronizing an aging brain: can entraining circadian clocks by food slow Alzheimer’s disease?. Front Aging Neurosci.

[57] Freitas P, Carvalho D, Santos AC, Madureira AJ, Martinez E (2014). Adipokines, hormones related to body composition and insulin resistance in HIV fat redistribution syndrome. BMC Infect Dis.

[58] Andersen O, Haugaard SB, Holst JJ, Deacon CF, versen J (2005). Enhanced glucagon-like peptide-1 (GLP-1) response to oral glucose in glucose-intolerant HIV-infected patients on antiretroviral therapy. HIV Med.

[59] Heaton RK, Franklin DR, Ellis RJ, McCutchan JA, Letendre SL (2011). HIV-associated neurocognitive disorders before and during the era of combination antiretroviral therapy: Differences in rates, nature and predictors. J Neurovirol.

[60] Bhaskaran K, Mussini C, Antinori A, Walker AS, Dorrucci M (2008). Changes in the incidence and predictors of human immunodeficiency virus-associated dementia in the era of highly active antiretroviral therapy. Ann Neurol.

[61] Bozzette SA, Ake CF, Tam HK, Chang SW, Louis TA (2003). Cardiovascular and cerebrovascular events in patients treated for human immunodeficiency virus infection. N Engl J Med.

[62] Currier J (2007). Report from the 14th retrovirus conference. Metabolic complications: lipoatrophy, lipohypertrophy and cardiovascular risk. AIDS Clin Care.

[63] Willig AL, Overton ET (2016). Metabolic complications and glucose metabolism in HIV infection: A review of the evidence. Curr HIV/AIDS Rep.

[64] Martin-Iguacel R, Negredo E, Peck R (2016). Hypertension is a key feature of the metabolic syndrome in subjects aging with HIV. Curr Hypertens Rep.

[65] Mirani G, Williams PL, Chernoff M, Abzug MJ, Levin MJ (2015). Changing trends in complications and mortality rates among US youth and young adults with HIV infection in the era of combination antiretroviral therapy. Clin Infect Dis.

[66] Becker JT, Kingsley L, Mullen J, Cohen B, Martin E (2009). Vascular risk factors, HIV serostatus and cognitive dysfunction in gay and bisexual men. Neurology.

[67] Wright EJ, Grund B, Robertson K, Brew BJ, Roediger M (2010). Cardiovascular risk factors associated with lower baseline cognitive performance in HIV-positive persons. Neurology.

[68] Mora-Peris B, Stevens E, Ferretti F, Underwood J, Taylor S (2016). Evolution of changes in cognitive function after the initiation of antiretroviral therapy. AIDS Res Ther.

[69] Ciccarelli N, Fabbiani M, Di Giambenedetto S, Fanti I, Baldonero E (2011). Efavirenz associated with cognitive disorders in otherwise asymptomatic HIV-infected patients. Neurology.

[70] Robertson KR, Su Z, Margolis DM, Krambrink A, Havlir DV (2010). Neurocognitive effects of treatment interruption in stable HIV-positive patients in an observational cohort. Neurology.

[71] Andrieu S, Aboderin I, Baeyens JP, Beard J, Benetos A (2011). IAGG workshop: Health promotion program on prevention of late onset dementia. J Nutr Health Aging.

[72] Koethe JR, Heimburger DC, PrayGod G, Filteau S (2016). From wasting to obesity: The contribution of nutritional status to immune activation in HIV infection. J Infect Dis.

[73] Gustafson DR (2010). Adiposity hormones and dementia. J Neurol Sci.

[74] Rothman KJ (1990). No adjustments are needed for multiple comparisons. Epidemiology.

